# ALA-Induced Flavonols Accumulation in Guard Cells Is Involved in Scavenging H_2_O_2_ and Inhibiting Stomatal Closure in *Arabidopsis* Cotyledons

**DOI:** 10.3389/fpls.2016.01713

**Published:** 2016-11-15

**Authors:** Yuyan An, Xinxin Feng, Longbo Liu, Lijun Xiong, Liangju Wang

**Affiliations:** College of Horticulture, Nanjing Agricultural UniversityNanjing, China

**Keywords:** abscisic acid (ABA), 5-aminolevulinic acid (ALA), flavonol, hydrogen peroxide, stomatal opening

## Abstract

5-aminolevulinic acid (ALA), a new plant growth regulator, can inhibit stomatal closure by reducing H_2_O_2_ accumulation in guard cells. Flavonols are a main kind of flavonoids and have been proposed as H_2_O_2_ scavengers in guard cells. 5-aminolevulinic acid can significantly improve flavonoids accumulation in plants. However, whether ALA increases flavonols content in guard cells and the role of flavonols in ALA-regulated stomatal movement remains unclear. In this study, we first demonstrated that ALA pretreatment inhibited ABA-induced stomatal closure by reducing H_2_O_2_ accumulation in guard cells of *Arabidopsis* seedlings. This result confirms the inhibitory effect of ALA on stomatal closure and the important role of decreased H_2_O_2_ accumulation in this process. We also found that ALA significantly improved flavonols accumulation in guard cells using a flavonol-specific dye. Furthermore, using exogenous quercetin and kaempferol, two major components of flavonols in *Arabidopsis* leaves, we showed that flavonols accumulation inhibited ABA-induced stomatal movement by suppressing H_2_O_2_ in guard cells. Finally, we showed that the inhibitory effect of ALA on ABA-induced stomatal closure was largely impaired in flavonoid-deficient *transparent testa4* (*tt4*) mutant. In addition, exogenous flavonols recovered stomatal responses of *tt4* to the wild-type levels. Taken together, we conclude that ALA-induced flavonol accumulation in guard cells is partially involved in the inhibitory effect of ALA on ABA-induced H_2_O_2_ accumulation and stomatal closure. Our data provide direct evidence that ALA can regulate stomatal movement by improving flavonols accumulation, revealing new insights into guard cell signaling.

## Introduction

Stomatal movements regulate plant gas exchange with the environment, and thus are critical for plant growth and development (Wang et al., 2014). Numerous environmental or hormone factors can trigger stomatal movement (Kim et al., 2010). Perhaps because the difficulty in balancing CO_2_ uptake and transpirational water loss, to date, most studies related to stomatal movements have focused on regulation of stomatal closure, while fewer studies have been made to enhance stomatal opening to increase plant photosynthesis. However, enhancing stomatal aperture to improve the uptake of CO_2_ by terrestrial plants is an increasingly important problem due to global climate changes (Wang et al., 2014). 5-Aminolevulinic acid (ALA), a natural plant growth regulator, is known to improve plant photosynthesis under both normal (Hotta et al., 1997) and stressful conditions (Hotta et al., 1998; Nishihara et al., 2003; Wang et al., 2004; Liu et al., 2011; Zhang et al., 2012; Ali et al., 2013b). Recent investigations showed that ALA did not affect stomata development, but significantly inhibited stomatal closing (An et al., 2016a), indicating increasing stomatal aperture is an important mechanism of ALA-mediated improvement of photosynthesis. Most importantly, ALA improved plant drought tolerance simultaneously (An et al., 2016a), further suggesting its great application potential in agriculture and forestry.

Among the regulators of stomatal movement, abscisic acid (ABA) is the most important hormone in provoking stomatal closing (Dodd, 2003; Tanaka et al., 2005). The molecular and cellular mechanisms involved in stomatal movements have been particularly well characterized in the context of ABA-induced stomatal closure (Li et al., 2006). Hydrogen peroxide (H_2_O_2_) is an important signaling molecule in plants and has been proved as an essential signal in ABA-induced stomatal closure (Pei et al., 2000; Rhee, 2006). We recently demonstrated that ALA treatment inhibited ABA-induced stomatal closure by reducing H_2_O_2_ accumulation in guard cells (An et al., 2016a). These results revealed the interaction between ALA and ABA in regulating stomatal movement and indicated a crucial function of H_2_O_2_ in this interaction. However, how ALA reduces ABA-induced H_2_O_2_ accumulation is largely unknown.

Hydrogen peroxide content in guard cells is tightly regulated by the generating and scavenging systems. Hydrogen peroxide in plant cells can be synthesized via several routes (Neill et al., 2002), and more than one source of H_2_O_2_ have also been proposed for guard cells. The enzymatic sources of H_2_O_2_ that have been revealed in guard cells include NADPH oxidase (Kwak et al., 2003; Desikan et al., 2006), peroxidases (He et al., 2011), copper amine oxidase (An et al., 2008), and polyamine oxidase (Hou et al., 2013). Among these, plasma membrane NADPH oxidase has been identified as a main source of ABA-induced H_2_O_2_ production in guard cells of *Arabidopsis* (Kwak et al., 2003). Comparatively, the scavenging mechanism of H_2_O_2_ in guard cells which suppresses stomatal closure has received little attention. Although it has been well documented that reactive oxygen species (ROS) in plant cells can be rapidly detoxified by various cellular enzymatic and small molecule antioxidants (Mittler et al., 2004), direct evidence on how H_2_O_2_ is scavenged in guard cells during stomatal opening is still lacking. Miao et al. (2006) showed that glutathione peroxidase 3 (AtGPX3) functioned as a ROS scavenger in ABA signaling. Munemasa et al. (2013) found that depletion of glutathione contributed to a higher level of ABA-induced H_2_O_2_ accumulation, indicating glutathione is also a H_2_O_2_ scavenger in ABA signaling. Our previous study suggested that ALA reduced H_2_O_2_ in guard cells mainly through accelerating its elimination (An et al., 2016a). However, until now, little is known about how ALA scavenges H_2_O_2_ in guard cells.

Many plant secondary metabolites act as antioxidants and can affect ROS concentrations (Chobot and Hadacek, 2011). Flavonoids are an important group of plant secondary metabolites that perform as antioxidants (Nakabayashi et al., 2014; Nguyen et al., 2016). Flavonols are among the most abundant flavonoids in plants (Winkel-Shirley, 2002; Martens et al., 2010). The flavonol branch pathway has remained intact for millions of years, and is almost exclusively involved in the responses of plants to a wide range of environmental stimuli (Pollastri and Tattini, 2011). Flavonols may act as defense molecules, signaling molecules, antioxidants, auxin transport inhibitors, and developmental regulators (Agati and Tattini, 2010; Pandey et al., 2015; Kuhn et al., 2016). Although flavonols have been well-documented for their antioxidant capacity *in vitro* (Yamasaki et al., 1997; Nakabayashi et al., 2014), their antioxidant capacity *in planta* is still a matter of controversy. In *Arabidopsis*, the main flavonoids in leaves are normally flavonols, including quercetin and kaempferol (Pelletier et al., 1999), and they accumulate mainly in the vacuoles of epidermal cells (Martens et al., 2010). Recently, Watkins et al. (2014) further demonstrated that in *Arabidopsis* flavonols accumulated specifically in guard cells and acted as a ROS scavenger in guard cells. 5-aminolevulinic acid can significantly improve flavonoids accumulation in fruits (Xie et al., 2013; Chen et al., 2015), leaves (Xu et al., 2011) and roots (Xu et al., 2010). However, no information is available on how ALA affects flavonols content in plants. We hypothesized that ALA may accelerate H_2_O_2_ removal by improving flavonols accumulation in guard cells and hence inhibit ABA-induced stomatal closure.

5-aminolevulinic acid pretreatment showed similar promotive effect on plant photosynthesis to concurrently applied ALA. However, whether ALA pretreatment also function through regulating stomatal movement remains unclear. Therefore, in this study, first, we investigated the effect of ALA pretreatment on stomatal movement and found that ALA pretreatment also inhibited ABA-induced stomatal closure by reducing H_2_O_2_ accumulation in guard cells. Then, using this experimental system and a flavonol-specific dye, we examined the effect of ALA on flavonols accumulation in guard cells and the influence of flavonols accumulation on stomatal movement. Furthermore, the role of flavonols accumulation in ALA-induced stomatal movement was investigated through a comparison of wild-type *Arabidopsis* plants and *transparent testa4* (*tt4*), the *Arabidopsis* chalcone synthase (CHS) mutant which is flavonoid-deficient. Our data provide direct evidence for ALA-mediated improvement of flavonols accumulation and demonstrate its positive role in ALA-induced stomatal movement, revealing new insights into guard cell signaling.

## Materials and Methods

### Plant Materials and Growth Conditions

*Arabidopsis* (*Arabidopsis thaliana*) of wild-type (Col-0, WT), ALA-over-producing transgenic lines (*YHem1*) (Zhang et al., 2010) and CHS *tt4* mutant that derived from Col-0 background were used in this study. Seeds were surface sterilized with bleaching power (5%, w/v) for 20 min, washed with sterilized water three times, then germinated and grown on Murashige and Skoog (MS) medium. Seedlings were cultured in a growth chamber at 25°C under a Photosynthetic Photo Flux Density (PPFD) of 150 μmol⋅m^-2^⋅s^-1^ in 12 h light/12 h dark cycles.

### Pretreatment with ALA, Exogenous Flavonol, and Treatment of ABA and H_2_O_2_

Two-week-old seedlings were used for experiments. Seedlings of WT, *YHem1*, and *tt4* with uniform height and size were carefully taken out of MS medium, washed off the medium, immersed in opening buffer (50 mM KCl, 10 mM MES, and 0.1 mM CaCl_2_, pH 6.2) and incubated under light conditions (PPFD 240 μmol m^-2^ s^-1^) for 2 h to open the stomata. These seedlings were then pretreated with ALA or exogenous flavonol before being exposed to ABA or H_2_O_2_. For ALA pretreatment, *YHem1* seedlings were immersed in opening buffer for another 4 h, and seedlings of WT or *tt4* were immersed in the opening buffer supplemented with 0.5 mg L^-1^ ALA for a further 4 h under light conditions. For exogenous flavonols pretreatment, seedlings with pre-opened stomata were immersed in the opening buffer supplemented with quercetin (0–100 μM) or kaempferol (0–100 μM) for a further 1 h under light conditions (PPFD 240 μmol m^-2^ s^-1^). For ABA or H_2_O_2_ treatment, pre-treated seedlings were immersed in the opening buffer supplemented with 10 μM ABA (Sigma-Aldrich, MO, USA) or 200 μM H_2_O_2_. Then, 10–12 seedlings of each treatment were randomly selected at 30-min intervals for 2 h and the cotyledons were picked with tweezers and used for stomatal bioassay and measurement of endogenous H_2_O_2_ and flavonol content. To avoid any potential rhythmic effects on stomatal aperture, experiments were always started at 8:00 am of the day.

### Stomatal Bioassay

Because the obtainment of suitable rosette leaves needs more than 4 weeks, to expedite the experimental process, the cotyledons from two-week-old seedlings were used for experiments in this study. Stomatal bioassay was performed directly on the cotyledons because cotyledons are thin and pale green, allowing us to easily observe stomatal movements without peeling off the epidermal tissues (Amodeo et al., 1996; Jiang et al., 2014; Shang et al., 2016). Stomatal apertures were observed by a light microscope (Nikon TE100, 400×), using a fitted camera (MShot Digital Imaging System), and measured with Adobe Photoshop 6.0 (Adobe systems, CA, USA). In each treatment, 30 randomly selected apertures were scored at each time point and experiments were repeated three times. The data presented are means of 90 measurements ± standard errors (SE).

### Measurement of Endogenous H_2_O_2_ and Flavonol Content Using Confocal Laser-Scanning Microscopy

Endogenous H_2_O_2_ was measured with the fluorescent indicator H_2_DCF-DA as described by An et al. (2016a). Seedlings after each treatment were placed into Tris-KCl buffer (10 mM Tris and 50 mM KCl, pH 6.5) containing H_2_DCF-DA (Sigma-Aldrich, USA) at 50 μM for 30 min, in the dark at 25°C. Endogenous flavonol was measured with the fluorescent indicator dye diphenylboric acid 2-aminoethyl ester (DPBA) as described by Watkins et al. (2014). After each treatment, seedlings were placed into Mes-KCl buffer (50 mM KCl, 10 mM MES, and 0.1 mM CaCl_2_, pH 6.2) containing 0.01% (v/v) Triton X-100 and DPBA (Sigma-Aldrich, USA) at 2.52 mg mL^-1^ for 30 min, in the dark at 25°C. After staining, excess H_2_DCF-DA and DPBA dye were removed with fresh Tris-KCl buffer and Mes-KCl buffer, respectively, in the dark. The cotyledons were picked with tweezers and their fluorescence was observed using a laser scanning confocal microscope (Leica TCS SP2, LSCM), with the following settings: ex = 488 nm, em = 525 ± 15 nm, power 50%, zoom 16, mild scanning, frame 512 × 512. Chlorophyll signal was collected between 650 to 700 nm. The DCF or DPBA fluorescence intensities were measured by placing a region of interest (ROI) around the guard cell. The intensity values within each ROI were recorded and averaged. In the determination of H_2_O_2_ and flavonol, at least 3 biological replicates were performed and 5 images were taken for each biological replicate.

### Statistical Analysis

All data were taken from at least three independent experiments. Statistical analysis was performed using SPSS statistical computer package (version 16.0 SPSS Inc. Chicago. IL). Data was compared with the control or among treatments by analysis of variance (ANOVA) to discriminate significant differences at *P*<0.05 or *P*<0.01 followed by least significant difference tests (LSD).

## Results

### ALA Pretreatment Inhibits ABA-Induced H_2_O_2_ Accumulation and Stomatal Closure

**FIGURE 1:**
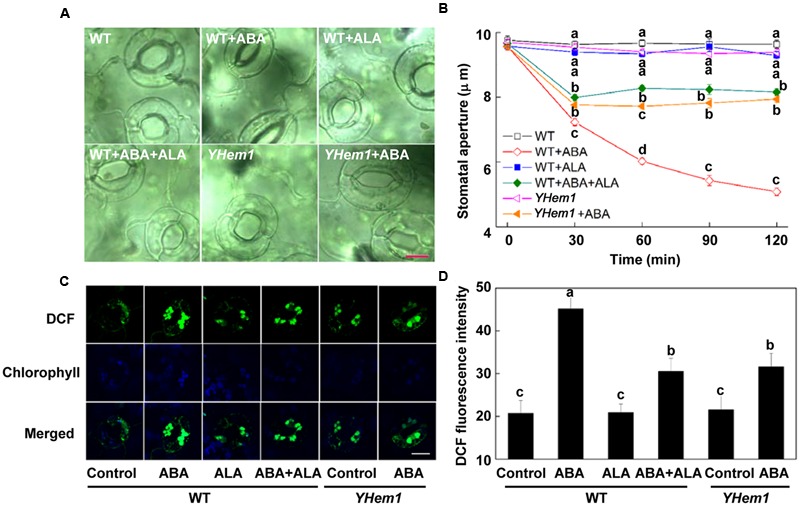
**ALA pretreatment inhibits ABA-induced stomatal closure by reducing H_2_O_2_ accumulation in guard cells. (A,B)** ALA pretreatment inhibits ABA-induced stomatal aperture. Two-week-old wild-type *Arabidopsis* pretreated with exogenous 0.5 mg L^-1^ ALA under light (240 μmol m^-2^ s^-1^) for 4 h and *YHem1*-transgenic *Arabidopsis* incubated under the same light condition for 4 h were immersed in opening buffer (50 mM KCl, 10 mM MES, and 0.1 mM CaCl_2_, pH 6.2) alone or containing 10 μM ABA for 2 h under light (240 μmol m^-2^ s^-1^). The cotyledons were picked with tweezers at 30-min intervals for 2 h for the record of guard cell images and determination of stomatal apertures. **(A)** Images of guard cell after 1 h of different treatments. Scale bar: 10 μm. **(B)** Time courses of stomatal responses in WT and *YHem1* to different treatments. Values are the means of 90 measurements ± SE from three independent experiments. Different letters on the same time point indicate significant differences at *P* = 0.05 level. **(C,D)** ALA pretreatment reduces ABA-induced H_2_O_2_ accumulation in guard cells. The cotyledons of the above treated seedlings were picked with tweezers at 1 h of treatment and loaded with 50 μM H_2_DCF-DA for 30 min in darkness at 25°C. Fluorescence of the cotyledons **(C)** was observed using a confocal microscope. For each treatment, DCF fluorescence is shown in green and chlorophyll autofluorescence is shown in blue in separate channels. Scale bar: 15 μm. **(D)** DCF intensity in guard cells of each treatment. Values are the means of 15 measurements ± SE from three independent experiments. Different letters indicate significant differences at *P* = 0.05 level.

Previously, we have demonstrated that ALA inhibits ABA-induced stomatal closure via down-regulation of H_2_O_2_ accumulation when ALA is applied together with ABA (An et al., 2016a). To further determine whether ALA pretreatment has the same effect, we compared stomatal responses in cotyledons of two-week-old *Arabidopsis* to ABA with or without ALA pretreatment for 4 h. ABA significantly reduced stomatal aperture of wild-type (WT) plants after 30 min and decreased it by almost 50% within 2 h (**Figures 1A,B**). When ABA was applied to *YHem1* (ALA-overproducing transgenic plants) or ALA-pretreated WT plants, ABA-induced stomatal closing was significantly suppressed. The time course for stomatal movement illustrated that the inhibition of ALA on ABA-induced stomatal closure was initiated after ABA application and lasted for at least 2 h (**Figure 1B**). These results indicate that ALA pretreatment inhibits ABA-induced stomatal closure.

To determine whether ALA pretreatment inhibits ABA-induced stomatal closure also by reducing ROS accumulation, we estimated the H_2_O_2_ content in guard cells using a fluorescent dye, H_2_DCF-DA. We found that ABA treatment for 1 h significantly increased H_2_O_2_ accumulation in guard cells of WT plants to more than two-fold of control plants. Without ABA application, the *YHem1* transgenic plants and ALA-pretreated WT plants showed the same level of DCF fluorescence intensity with non-pretreated WT plants (**Figures 1C,D**). When ABA was applied to *YHem1* or ALA-pretreated WT plants, ABA-induced H_2_O_2_ accumulation was suppressed by about 35%. These results indicate that, similar to the effect of ALA treatment, ALA pretreatment also reduces ABA-induced H_2_O_2_ accumulation and then inhibits stomatal closure.

### ALA Pretreatment Increases Flavonols Accumulation in Guard Cell

**FIGURE 2:**
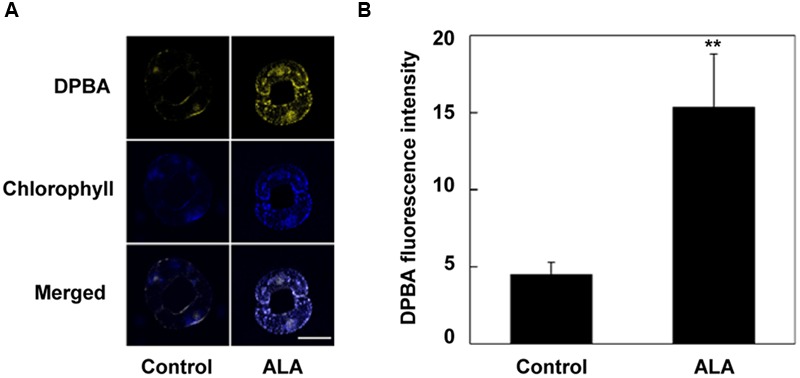
**ALA pretreatment increases flavonols accumulation in guard cells.** Two-week-old wild-type (WT) *Arabidopsis* pretreated with exogenous 0.5 mg L^-1^ ALA under light (240 μmol m^-2^ s^-1^) for 4 h and *YHem1*-transgenic *Arabidopsis* incubated under the same light condition for 4 h were loaded with 2.52 mg mL^-1^ diphenylboric acid 2-aminoethyl ester (DPBA) for 30 min in darkness at 25°C. The cotyledons were then picked with tweezers and their fluorescence **(A)** was observed using a confocal microscope. For each treatment, DPBA bound to flavonols is shown in yellow and chlorophyll autofluorescence is shown in blue in separate channels. Scale bar: 15 μm. **(B)** DPBA intensity values in guard cells of each treatment. Values are the means of 15 measurements ± SE from three independent experiments. ^∗∗^ indicates significant differences between treatments at *P* = 0.01 level.

A role of flavonols in suppressing H_2_O_2_ accumulation and stomatal closure has been suggested (Watkins et al., 2014). To determine whether flavonols accumulation contributes to ALA-mediated reduction of H_2_O_2_ content, we first investigated the effect of ALA on flavonols content in guard cells using a flavonol-specific fluorescent dye, DPBA. We found that ALA pretreatment for 4 h significantly increased flavonols accumulation (**Figure 2**). The DPBA fluorescence intensity in guard cells of ALA-pretreated plants was about 2.44-fold higher than that in control plants.

### Flavonols Inhibit ABA-Induced Stomatal Closure

**FIGURE 3:**
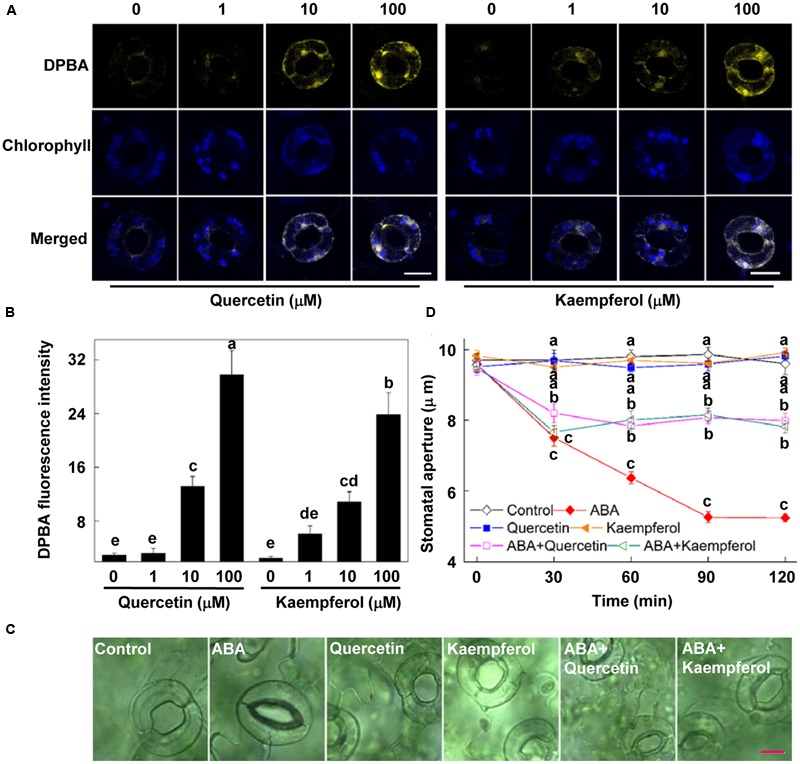
**Flavonols inhibit ABA-induced stomatal closure. (A,B)** Exogenous flavonols increase endogenous flavonols accumulation in guard cells. Two-week-old wild-type *Arabidopsis* were immersed in opening buffer (50 mM KCl, 10 mM MES, and 0.1 mM CaCl_2_, pH 6.2) alone or containing 1–100 μM quercetin or kaempferol for 1 h under light (240 μmol m^-2^ s^-1^) and then loaded with 2.52 mg mL^-1^ DPBA for 30 min in darkness at 25°C. The cotyledons were then picked with tweezers and their fluorescence **(A)** was observed using a confocal microscope. For each treatment, DPBA bound to flavonols is shown in yellow and chlorophyll autofluorescence is shown in blue in separate channels. Scale bar: 15 μm. **(B)** DPBA intensity in guard cells of each treatment. Values are the means of 15 measurements ± SE from three independent experiments. The same letters indicate no significant differences at *P* = 0.05 level. **(C,D)** Flavonols inhibit ABA-induced stomatal closure. Two-week-old wild-type *Arabidopsis* pretreated with exogenous 10 μM quercetin or kaempferol for 1 h under light (240 μmol m^-2^ s^-1^) were immersed in opening buffer (50 mM KCl, 10 mM MES, and 0.1 mM CaCl_2_, pH 6.2) alone or containing 10 μM ABA for 2 h under light (240 μmol m^-2^ s^-1^). The cotyledons were picked with tweezers at 30-min intervals for 2 h for the record of guard cell images and determination of stomatal apertures. **(C)** Images of guard cell after 1 h of different treatments. Scale bar: 10 μm. **(D)** Time courses of stomatal responses to different treatments. Values are the means of 90 measurements ± SE from three independent experiments. Different letters on the same time point indicate significant differences at *P* = 0.05 level.

To determine whether flavonols accumulation in guard cells can regulate stomatal movement, we investigated the effects of exogenous quercetin and kaempferol, two major components of flavonol in *Arabidopsis* leaves, on ABA-induced stomatal closure. First, to choose the appropriate concentration of exogenous flavonol, we detected the flavonols content in guard cells after treatment with quercetin or kaempferol of different concentrations for 1 h. Results showed that the concentrations equal to or higher than 10 μM of quercetin and kaempferol significantly increased endogenous flavonols content (**Figures 3A,B**). Since flavonols content in guard cells of plants treated with 10 μM quercetin and kaempferol was similar to that in ALA-pretreated plants (**Figures 2** and **3A,B**), we choose 10 μM quercetin and kaempferol for the following experiments.

Stomatal bioassays showed that quercetin or kaempferol alone did not influence stomatal aperture (**Figures 3C,D**). When applied together with ABA, both of them significantly suppressed ABA-induced stomatal closure, compared to the effect of ABA alone. After being treated for 2 h, stomatal aperture of plants treated with ABA and quercetin or kaempferol was about 50% higher than that of plants treated with ABA alone (**Figure 3D**). These results demonstrated that flavonols accumulation in guard cells inhibited ABA-induced stomatal closure, indicating that flavonols might play an important role in the inhibition of ABA-induced stomatal closure by ALA.

### Flavonols decrease H_2_O_2_ Accumulation in Guard Cells and Inhibit H_2_O_2_-Induced Stomatal Closure

**FIGURE 4:**
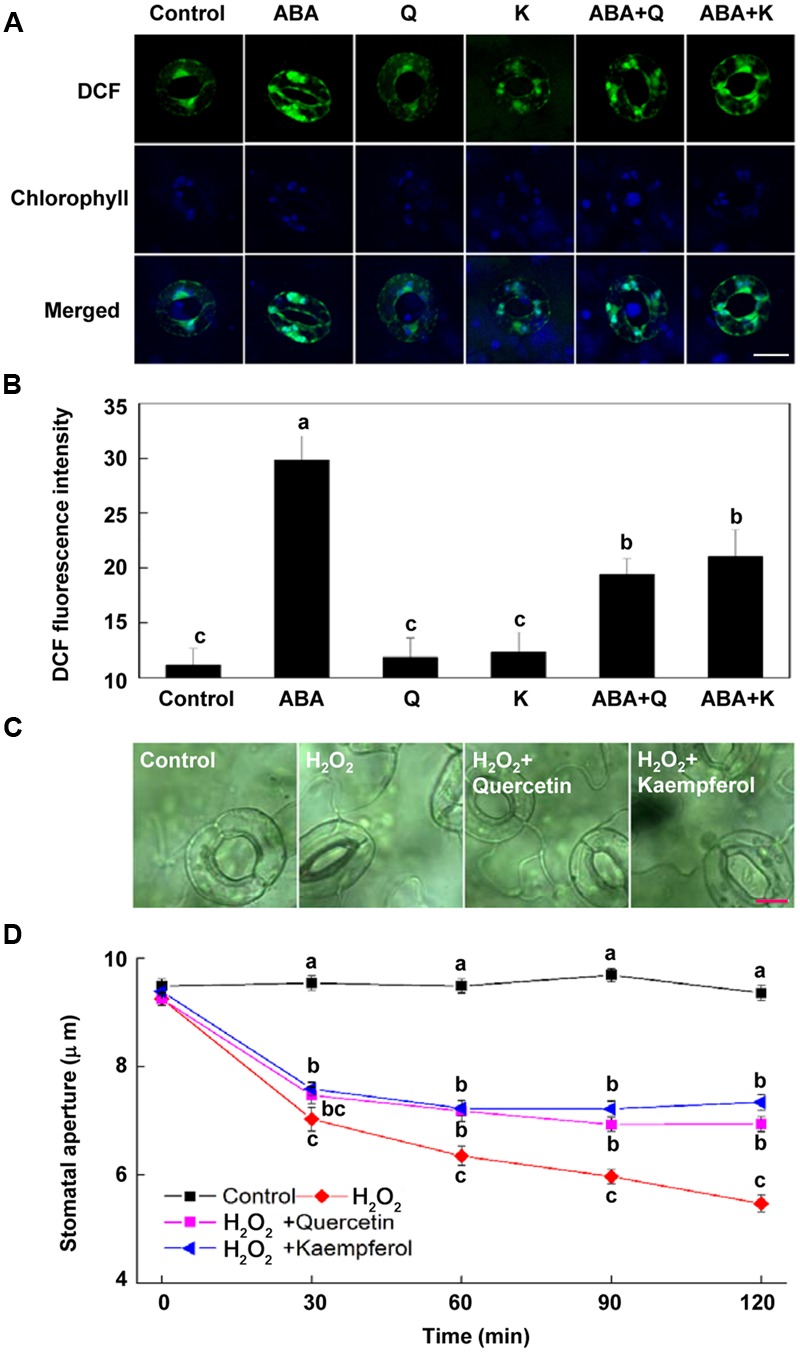
**Flavonols reduce H_2_O_2_ accumulation in guard cells. (A,B)** Flavonols reduce ABA-induced H_2_O_2_ accumulation in guard cells. Two-week-old wild-type *Arabidopsis* pretreated with exogenous 10 μM quercetin (Q) or kaempferol (K) for 1 h under light (240 μmol m^-2^ s^-1^) were immersed in opening buffer (50 mM KCl, 10 mM MES, and 0.1 mM CaCl_2_, pH 6.2) alone or containing 10 μM ABA for 1 h under light (240 μmol m^-2^ s^-1^), and then loaded with 50 μM H_2_DCF-DA for 30 min in darkness at 25°C. The cotyledons were picked with tweezers and their fluorescence **(A)** was observed using a confocal microscope. For each treatment, DCF fluorescence is shown in green and chlorophyll autofluorescence is shown in blue in separate channels. Scale bar: 15 μm. **(B)** DCF intensity in guard cells of each treatment. Values are the means of 15 measurements ± SE from three independent experiments. Different letters indicate significant differences at *P* = 0.05 level. **(C,D)** Flavonols inhibit H_2_O_2_-induced stomatal closure. Two-week-old *Arabidopsis* pretreated with exogenous 10 μM quercetin or kaempferol for 1 h under light (240 μmol m^-2^ s^-1^) were immersed in opening buffer (50 mM KCl, 10 mM MES, and 0.1 mM CaCl_2_, pH 6.2) alone or containing 200 μM H_2_O_2_ for 2 h under light (240 μmol m^-2^ s^-1^). The cotyledons were picked with tweezers at 30-min intervals for 2 h for the record of guard cell images and determination of stomatal apertures. **(C)** Images of guard cell after 1 h of different treatments. Scale bar: 10 μm. **(D)** Time courses of stomatal responses to different treatments. Values are the means of 90 measurements ± SE from three independent experiments. Different letters on the same time point indicate significant differences at *P* = 0.05 level.

To determine whether flavonol accumulation inhibits ABA-induced stomatal closure by reducing H_2_O_2_ content in guard cells, we investigated the effects of exogenous flavonols on H_2_O_2_ content in guard cells. We found that both quercetin and kaempferol significantly decreased ABA-induced H_2_O_2_ accumulation in guard cells (**Figures 4A,B**), consistent with their effects on flavonols accumulation. These results indicate that flavonols accumulation reduces H_2_O_2_ content and consequently suppresses ABA-induced stomatal closure.

To confirm the effect of flavonols accumulation on H_2_O_2_ content and its role in stomatal closure, we evaluated whether flavonols inhibited H_2_O_2_-induced stomatal closure. Results showed that both quercetin and kaempferol significantly suppressed H_2_O_2_-induced stomatal closure (**Figures 4C,D**). After being treated for 2 h, stomatal aperture of plants treated with H_2_O_2_ and quercetin or kaempferol was about 30% higher than that of plants treated with H_2_O_2_ alone (**Figure 4D**). These results confirmed that flavonols accumulation could scavenge H_2_O_2_ in guard cells and inhibit stomatal closure.

### Flavonols Accumulation Involves in ALA-Regulated Stomatal Movement

**FIGURE 5:**
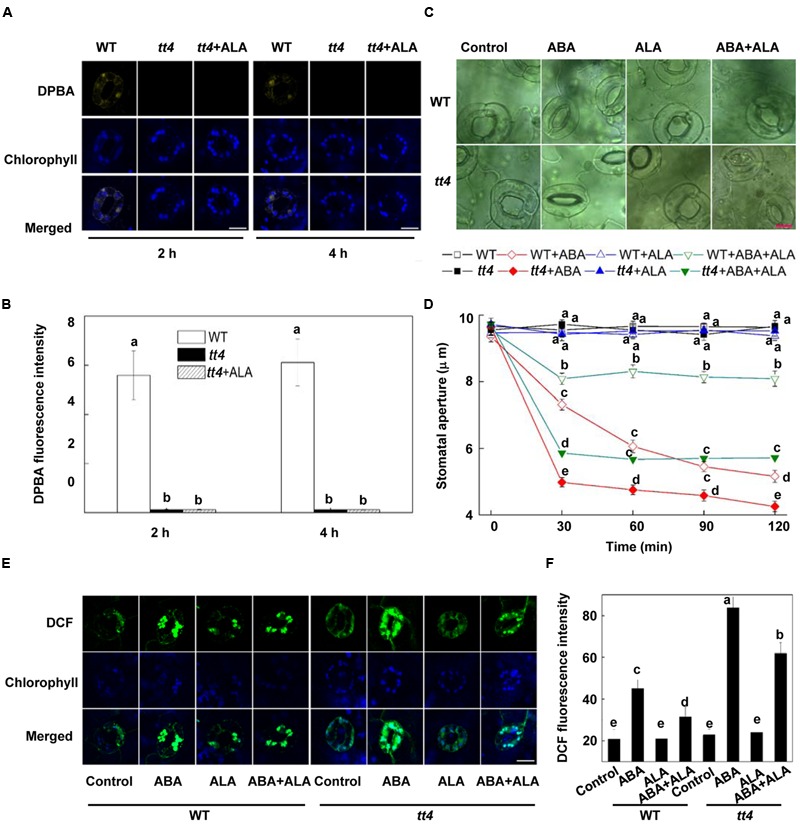
**The inhibitory effect of ALA on ABA-induced stomatal closure is impaired in *tt4* mutant. (A,B)** Flavonols accumulation is absent in *tt4* mutant pretreated with or without ALA. Two-week-old wild-type and *tt4 Arabidopsis* pretreated with exogenous 0.5 mg L^-1^ ALA under light (240 μmol m^-2^ s^-1^) for 2 h or 4 h were loaded with 2.52 mg mL^-1^ DPBA for 30 min in darkness at 25°C. The cotyledons were then picked with tweezers and their fluorescence **(A)** was observed using a confocal microscope. For each treatment, DPBA bound to flavonols is shown in yellow and chlorophyll autofluorescence is shown in blue in separate channels. Scale bar: 15 μm. **(B)** DPBA intensity in guard cells of each treatment. Values are the means of 15 measurements ± SE from three independent experiments. Different letters indicate significant differences at *P* = 0.01 level. **(C,D)** The inhibition of ABA-induced stomatal closure by ALA is impaired in *tt4* mutant. Two-week-old wild-type and *tt4 Arabidopsis* pretreated with exogenous 0.5 mg L^-1^ ALA under light (240 μmol m^-2^ s^-1^) for 4 h were immersed in opening buffer (50 mM KCl, 10 mM MES, and 0.1 mM CaCl_2_, pH 6.2) alone or containing 10 μM ABA for 2 h under light (240 μmol m^-2^ s^-1^). The cotyledons were picked with tweezers at 30-min intervals for 2 h for the record of guard cell images and determination of stomatal apertures. **(C)** Images of guard cell after 1 h of different treatments. Scale bar: 10 μm. **(D)** Time courses of stomatal responses in WT and *tt4* to different treatments. Values are the means of 90 measurements ± SE from three independent experiments. Different letters on the same time point indicate significant differences at *P* = 0.05 level. **(E,F)** The inhibition of ABA-induced H_2_O_2_ accumulation in guard cells by ALA is impaired in *tt4* mutant. The cotyledons of the seedlings treated as shown in Figures C and D were picked at 1 h of treatment and loaded with 50 μM H_2_DCF-DA for 30 min in darkness at 25°C. Fluorescence of the cotyledons **(E)** was observed using a confocal microscope. For each treatment, DCF fluorescence is shown in green and chlorophyll autofluorescence is shown in blue in separate channels. Scale bar: 15 μm. **(F)** DCF intensity in guard cells of each treatment. Values are the means of 15 measurements ± SE from three independent experiments. Different letters indicate significant differences at *P* = 0.05 level.

The above results indicate that ALA-induced flavonols accumulation mediates ALA’s inhibitory effects on H_2_O_2_ accumulation and stomatal closure. To verify the role of flavonols accumulation, we investigated the effect of ALA on ABA-induced stomatal closure using the *transparent testa 4* (*tt4*) *Arabidopsis* mutant that makes no flavonoids (Nakabayashi et al., 2014). **Figures 5A,B** showed that flavonols did not accumulate in the *tt4* mutant, even with the application of exogenous ALA. The *tt4* mutant showed similar stomatal aperture to WT plants under control conditions (**Figures 5C,D**). ABA significantly reduced stomatal aperture of WT and *tt4*. However, stomatal response of *tt4* to ABA was significantly faster and greater than that of WT. Stomatal aperture of *tt4* was 31.97% and 17.61% lower than that of WT after 30 min and 120 min of ABA treatment, respectively. When ABA was applied together with ALA, ABA-induced stomatal closure was significantly inhibited by ALA in either WT or *tt4*. However, compared with WT plants, *tt4* showed significantly greater ABA-induced stomatal closure response when ABA and ALA were applied together. Stomatal aperture of *tt4* was about 30% lower than that of WT at each time point under ABA+ALA treatment. Measurement of H_2_O_2_ content showed that, compared with their corresponding control, ABA significantly increased H_2_O_2_ content in the guard cells of both WT and *tt4*, and the H_2_O_2_ accumulation in *tt4* was greater than that in WT (**Figures 5E,F**). When ABA was applied together with ALA, ABA-induced H_2_O_2_ accumulation was significantly decreased. Furthermore, compared with WT, *tt4* showed remarkably higher H_2_O_2_ content when ABA and ALA were applied together. These results indicate that ALA inhibits ABA-induced stomatal closure in both WT and *tt4* by decreasing ROS accumulation in guard cells, but this inhibitory effect of ALA is impaired in *tt4* mutant, compared to WT. Our data verified that flavonols accumulation played an important role in ALA-regulated stomatal movement.

**FIGURE 6:**
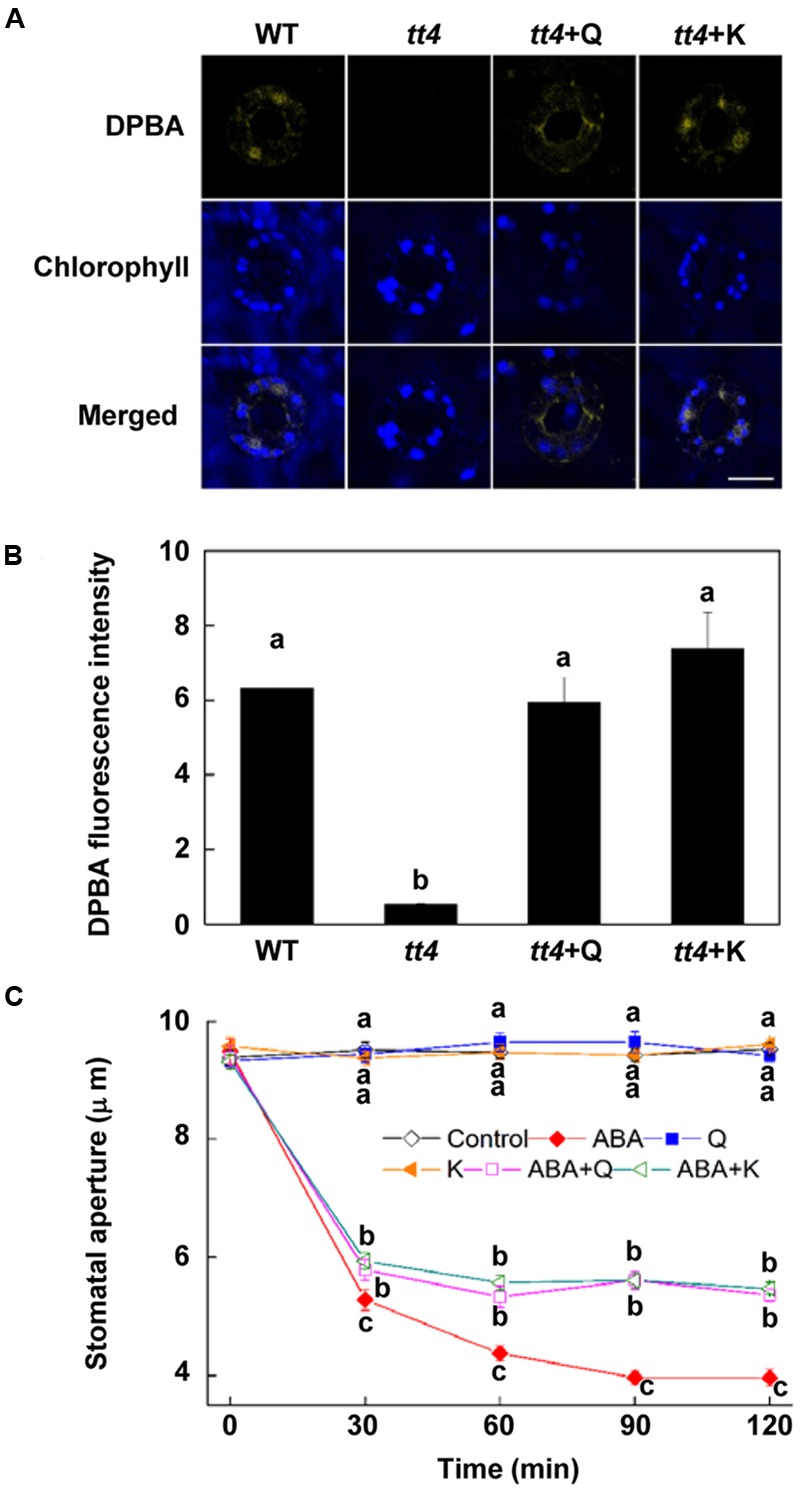
**Stomatal response of *tt4* to ABA is recovered by exogenous flavonols. (A,B)** Flavonols accumulation in *tt4* is recovered to the WT level by exogenous flavonols. Two-week-old wild-type and *tt4 Arabidopsis* pretreated with or without exogenous 10 μM quercetin (Q) or kaempferol (K) under light (240 μmol m^-2^ s^-1^) for 1 h were loaded with 2.52 mg mL^-1^ DPBA for 30 min in darkness at 25°C. The cotyledons were then picked with tweezers and their fluorescence **(A)** was observed using a confocal microscope. For each treatment, DPBA bound to flavonols is shown in yellow and chlorophyll autofluorescence is shown in blue in separate channels. Scale bar: 15 μm. **(B)** DPBA intensity in guard cells of each treatment. Values are the means of 15 measurements ± SE from three independent experiments. Different letters indicate significant differences at *P* = 0.01 level. **(C)** Stomatal response of *tt4* to ABA is recovered by exogenous flavonols. Two-week-old *tt4 Arabidopsis* pretreated with exogenous 10 μM quercetin (Q) or kaempferol (K) under light (240 μmol m^-2^ s^-1^) for 1 h were immersed in opening buffer (50 mM KCl, 10 mM MES, and 0.1 mM CaCl_2_, pH 6.2) alone or containing 10 μM ABA for 2 h under light (240 μmol m^-2^ s^-1^). The cotyledons were picked with tweezers at 30-min intervals for 2 h for the record of guard cell images and determination of stomatal apertures. Values are the means of 90 measurements ± SE from three independent experiments. Different letters on the same time point indicate significant differences at *P* = 0.05 level.

**FIGURE 7:**
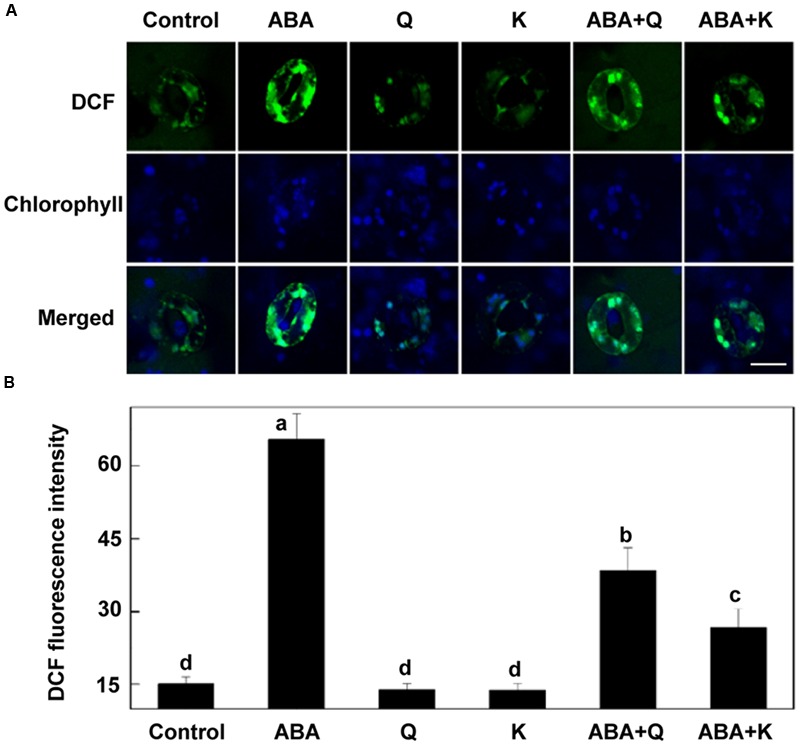
**H_2_O_2_ accumulation in *tt4* under ABA treatment is recovered by exogenous flavonols.** Two-week-old *tt4* mutants pretreated with or without exogenous 10 μM quercetin (Q) or kaempferol (K) under light (240 μmol m^-2^ s^-1^) for 1 h were loaded with 50 μM H_2_DCF-DA for 30 min in darkness at 25°C. The cotyledons were then picked with tweezers and their fluorescence **(A)** was observed using a confocal microscope. For each treatment, DCF fluorescence is shown in green and chlorophyll autofluorescence is shown in blue in separate channels. Scale bar: 15 μm. **(B)** DCF intensity in guard cells of each treatment. Values are the means of 15 measurements ± SE from three independent experiments. Different letters indicate significant differences at *P* = 0.01 level.

To further confirm the role of flavonols accumulation, we detected whether exogenous flavonols could restore stomatal responses of *tt4* mutant to ABA and ALA. Results showed that 10 μM quercetin or kaempferol increased the endogenous flavonols content in *tt4* guard cells to the WT level (**Figures 6A,B**). Although ABA-induced stomatal closure response was greater in *tt4* mutant than in control plants, exogenous flavonols induced stomatal responses of *tt4* recoverd to the WT level (**Figures 5D** and **6C**). After exposure to ABA for 2 h, the stomatal aperture of *tt4* was 23.26% lower than that of WT, however, it recovered to about 105% of that of WT when exogenous flavonols were applied concurrently (**Figures 5D** and **6C**). We further demonstrated that exogenous flavonols reduced H_2_O_2_ accumulation in *tt4* to nearly WT levels (**Figures 4A,B** and **7**). These results confirmed the important role of flavonols in scavenging H_2_O_2_ and then inhibiting stomatal closure.

## Discussion

It has been well-documented that ALA, a new plant growth regulator, significantly improves plant photosynthesis and growth (Akram and Ashraf, 2013). However, its underlying mechanisms remain largely unknown. Stomatal aperture is a major limiting factor in photosynthesis and plant growth (Lawson et al., 2014; Wang et al., 2014). Our recent study showed that ALA significantly inhibited stomatal closing (An et al., 2016a), indicating ALA-induced stomatal opening is an important reason for the improvement of photosynthesis. Here, we demonstrated that ALA pretreatment also significantly inhibited ABA-induced stomatal closing. These results are consistent with previous reports that both ALA treatment (Hotta et al., 1997; Nishihara et al., 2003; Tian et al., 2014) and pretreatment (Liu et al., 2011; Zhang et al., 2012; An et al., 2016b) universally improved plant photosynthesis. Although stomatal opening will inevitably increase transpirational water loss, which might decrease plant drought tolerance, several studies have shown that ALA largely improve drought tolerance of various plant species (Li et al., 2011; Liu et al., 2011, 2013; Kosar et al., 2015). Our previous study provides direct evidence that ALA inhibits stomatal closing but significantly improves plant drought tolerance simultaneously (An et al., 2016a). Increasing stomatal aperture indeed accelerates transpirational water loss, but transpiration simultaneously enhances water and nutrient uptake into plants, which is vital to plant growth. It has been reported that ALA significantly increased uptake of several nutrients, such as phosphate (Yao et al., 2006; Ali et al., 2014), nitrogen (Wei et al., 2012; Ali et al., 2014), sulfur (Ali et al., 2014), potassium calcium, magnesium, iron, copper, and zinc (Naeem et al., 2010; Zhang et al., 2015). 5-aminolevulinic acid also widely improves plant root activity and root growth (Kosar et al., 2015; An et al., 2016a,b), which is important for water and nutrient uptake and hence for plant growth and stress resistance (Sharp et al., 2003; Schachtman and Goodger, 2008). Therefore, the higher plant photosynthesis and the stronger root water/nutrient uptake induced by ALA are important mechanisms underlying ALA-improved drought tolerance. Although the exact mechanisms behind the paradox between enhanced drought tolerance and promoted stomatal opening still remain elusive, the obtained results suggest the great application potential of ALA in agriculture and forestry for a sustainable low-carbon society.

5-aminolevulinic acid not only increases plant drought tolerance but also can improve plant tolerance to various other abiotic stresses, such as salt (Nishihara et al., 2003), cold (Hotta et al., 1998), heat (Zhang et al., 2012), low light (Wang et al., 2004), and heavy metal stresses (Ali et al., 2013a; Tian et al., 2014). A common mechanism behind ALA’s function is the improvement of antioxidant capacity and the reduction of ROS accumulation. Guard cells represent a powerful single-cell model system for understanding signal transduction mechanisms in plants (Munemasa et al., 2015). Our recent research showed that ALA significantly reduced H_2_O_2_ content in guard cells and consequently inhibited stomatal closure (An et al., 2016a). Here, we further showed that ALA pretreatment also significantly reduced H_2_O_2_ accumulation in guard cells and inhibited stomatal closure. Together, these results indicate that ALA-mediated H_2_O_2_ decreasing can last for a long time. In guard cells, it contributes to ALA-induced stomatal opening, and in plant organs, such as leaves and roots, it helps decreasing abiotic stress-induced oxidative damage in plant tissues.

Hydrogen peroxide is an important second messenger in ABA signaling in guard cells (Pei et al., 2000). Its content in guard cells is tightly regulated by the production and scavenging systems. ABA-induced H_2_O_2_ accumulation is dependent on H_2_O_2_ synthesis in *Arabidopsis*, and the NADPH oxidase subunits, RESPIRATORY BURST OXIDASE HOMOLOG D and F (*Atr*bohD and *Atr*bohF, respectively) are required for the production of H_2_O_2_ during ABA-induced stomatal closure in guard cells (Kwak et al., 2003). However, recent research indicates that ALA-caused decrease of H_2_O_2_ in guard cells is not primarily through the inhibition of H_2_O_2_ synthesis but mainly through accelerating its elimination (An et al., 2016a). How ALA removes H_2_O_2_ in guard cells remains unclear. In this work, we provide evidence that ALA-induced flavonol accumulation plays an important role as a H_2_O_2_ scavenger in ALA-mediated stomatal movement in *Arabidopsis*. The following results support this conclusion: (i) ALA stimulated flavonols accumulation in guard cells; (ii) exogenous quercetin and kaempferol, two main kinds of flavonols in *Arabidopsis*, resulted in flavonols accumulation in guard cells and inhibited ABA- and H_2_O_2_-induced stomatal closure by decreasing H_2_O_2_ in guard cells; (iii) the inhibition of ABA-induced H_2_O_2_ accumulation and stomatal closure by ALA were impaired in *tt4* mutant which is flavonol deficient; (iv) exogenous quercetin and kaempferol restored the stomatal responses of *tt4* mutant to ABA. Watkins et al. (2014) first reported the signaling role of flavonol accumulation in ethylene-induced stomatal movement. Our data confirm the role of flavonol as a H_2_O_2_ scavenger in guard cell signaling. Although flavonols have been proposed as natural antioxidants (Nakabayashi et al., 2014; Nguyen et al., 2016), its antioxidant capacity *in planta* has long been debated. Our data together with report of Watkins et al. (2014) provide direct evidence that flavonols can scavenge H_2_O_2_ in guard cells, confirming its antioxidant capacity *in planta* and revealing new components in guard cell signaling. However, in the present study, ALA’s effects on H_2_O_2_ accumulation and stomatal closure were not totally inhibited, but just partially impaired in *tt4* mutant. These results indicate that the inhibitory effect of ALA pretreatment on ABA-induced stomatal closure is not completely dependent on flavonols accumulation, and further studies are needed to reveal other factors which also involve in this process.

Flavonols are ubiquitous phenolic plant secondary metabolites (Thompson et al., 2010). Previous studies in the past decades have indicated that ALA can stimulate phenylpropanoid metabolism and improve flavonoid accumulation in plants (Xu et al., 2010, 2011; Xie et al., 2013; Chen et al., 2015). However, no specific information is available on the effect of ALA on flavonols accumulation in plants. In the present study, using a flavonol-specific dye, we proved that ALA significantly improved flavonols accumulation in guard cells in *Arabidopsis*, providing direct evidence of ALA-stimulated flavonol accumulation in plants. Flavonols are involved in various physiological processes in plants including growth, development, and defense response to various stresses (Agati and Tattini, 2010; Pandey et al., 2015; Kuhn et al., 2016). Nakabayashi et al. (2014) suggest that flavonoid over-accumulation can enhance tolerance to multiple stresses. As mentioned earlier, improving antioxidant capacity was a common mechanism behind ALA-conferred plant tolerance to various stresses. Here, we demonstrated that ALA-induced flavonols accumulation decreases H_2_O_2_ content in guard cells. Therefore, flavonols accumulation may play important roles in ALA-induced improvement of plant antioxidant capacity. Besides their roles in plant biology, flavonols have been reported to provide several health benefits to mankind owing to their antioxidant and anti-inflammatory properties (Lee et al., 2015). Moreover, consumption of flavonol-rich food is helpful in lowering the risk of certain diseases (Lea, 2015; Pandey et al., 2015). For example, quercetin and kaempferol have synergistic actions in inhibiting cancer cell proliferation (Arima et al., 2002). Taken together, flavonols not only play important roles *in planta* but also have a close connection with human health. How to improve the flavonols content in plant has become a hot research topic in the field of biology (Jia et al., 2015). In this work, we prove that ALA stimulates flavonols accumulation in guard cells, indicating that application of ALA would be a potential method to improve flavonols in leaves of plants. Further studies will be carried out to elucidate the mechanisms how ALA improves the flavonols content in plants, which is largely unknown.

In summary, the data presented here demonstrate that ALA significantly enhances flavonols content in guard cells, and the flavonols accumulation contributes to the inhibitory effect of ALA on ABA-induced stomatal closure by suppressing H_2_O_2_ accumulation in guard cells. In flavonol-deficient *tt4* mutant, the inhibitory effect of ALA on ABA-induced H_2_O_2_ accumulation and stomatal closure was not completely suppressed, indicating that ALA-induced stomatal movement is not totally dependent on flavonols accumulation and there should be other pathways also involved in this physiological process. Our results provide new insights into the signal transduction pathway of ALA-induced stomatal movement, and add new potential method to improve plant flavonol content which shows multiple beneficial effects on plant itself and our humankind. The molecular mechanism how ALA regulates flavonol synthesis in plants is worthy of further study.

## Author Contributions

YA and LW conceived and designed research. YA, XF, LL, and LX carried out all the experiments. YA and XF analyzed the data. YA and LW wrote the manuscript. All authors read and approved the manuscript.

## Conflict of Interest Statement

The authors declare that the research was conducted in the absence of any commercial or financial relationships that could be construed as a potential conflict of interest.
